# Molecular epidemiology of dengue fever outbreaks in Bhutan, 2016-2017

**DOI:** 10.1371/journal.pntd.0008165

**Published:** 2020-04-22

**Authors:** Sangay Zangmo, Jit Bdr Darnal, Tsheten, Sonam Gyeltshen, Binay Thapa Thapa, Prinyada Rodpradit, Piyawan Chinnawirotpisan, Wudtichai Manasatienkij, Louis R. Macareo, Stefan Fernandez, Sonam Wangchuk, Chonticha Klungthong

**Affiliations:** 1 Royal Centre for Disease Control, Ministry of Health, Thimphu, Bhutan; 2 Department of Virology, Armed Forces Research Institute of Medical Sciences, Bangkok, Thailand; Stanford University, UNITED STATES

## Abstract

Dengue continues to pose a significant public health problem in tropical and subtropical countries. In Bhutan, first outbreak of dengue fever (DF) was reported in 2004 in a southern border town, followed by sporadic cases over the years. In this study, we analysed DF outbreaks that occurred in 3 different places during the years 2016 and 2017. A total of 533 cases in 2016 and 163 in 2017 were suspected of having of DF, where young adults were mostly affected. A total of 240 acute serum specimens collected and analyzed for serotype by nested RT-PCR revealed predominance of serotypes 1 and 2 (DENV-1 and 2). Phylogenetic analysis using envelope gene for both the serotypes demonstrated cosmopolitan genotype which were closely related to strains from India, indicating that they were probably imported from the neighboring country over the past few years.

## Author summary

Endemicity of DENV in some places of southern Bhutan has been established previously. In this study, we analysed outbreaks of DF that occurred in 3 places over a period of 2 years, 1 of which was previously not known to be endemic to DENV. Serum specimens collected from patients suspected of having DF were analyzed in the Royal Centre for Disease Control (RCDC) in Bhutan and in Armed Forces Research Institute of Medical Sciences (AFRIMS), Thailand. DENV-1 and 2 were established as the causes of the outbreaks, also indicating that serotypes of DENV circulating in the country over the past years have remained the same. Our analyses reveal that the current DENV-1and DENV-2 in Bhutan probably originated from India, Bhutan’s closest neighboring country.

## Introduction

Dengue virus (DENV), the etiological agent of dengue fever (DF) is a single, positive-stranded RNA virus of the genus *Flavivirus* under family *Flaviviridae*. DENV is prevalent in four genetically and antigenically different serotypes, DENV-1 to 4 [1,2]. Infection by a particular serotype confers long-lasting homotypic immunity to that serotype, but only leads to transient cross-protective immunity to a different serotype. Heterotypic antibodies often lead to dengue hemorrhagic fever (DHF) and dengue shock syndrome (DSS), the severe forms of dengue [3].

DF continues to be one of the most common vector borne diseases in tropical and sub-tropical regions of the world [4,5]. Every year, around 390 million people are infected globally, of which around 96 million manifest clinically with varying severity [5]. South East (SE) Asia and Western Pacific countries represent about 75% of the burden, and epidemics are experienced every 3–5 years in these regions [6].

**Fig 1:**
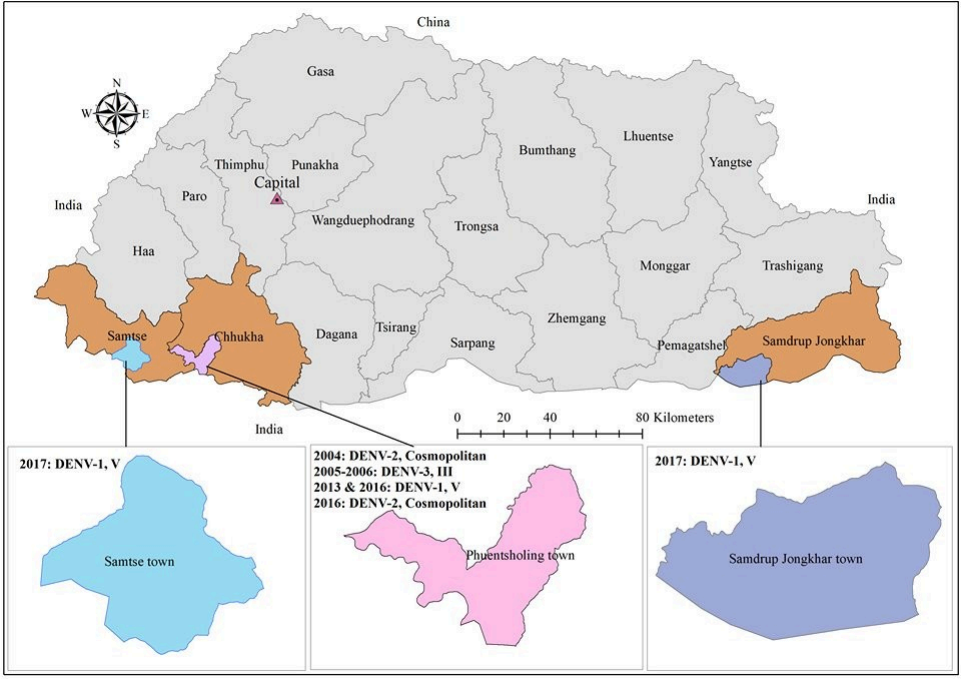
Map of Bhutan depicting places from where outbreaks of DF were reported. Year wise detection of DENV serotypes and their genotypes are provided alongside the place of outbreak. This map was created using ESRI, ArcGIS 10.5; 2016.

Bhutan is a land-locked Himalayan country lying at a height ranging from about 300 to 25,000 ft above sea level, It borders India to the east, west and south; and Tibet, China’s autonomous region to the north. India, Bhutan’s closest neighbor, reported dengue as early as 1946, and continues to exhibit a complex epidemiology with co-circulation of all 4 serotypes [7,8]. Dengue in Bhutan, was reported only in 2004 following an outbreak in the southern town of Phuntsholing under Chukha district [9]. Owing to the ever increasing urbanization along with international travel and trade, *Aedes* vectors have spread to newer geographical regions, leading to more frequent incidences of dengue in otherwise low risk areas [10]. In Bhutan, although both *Aedes aegypti* and *Aedes albopictus* were prevalent in all the seven southern districts, dengue remained endemic in the town of Phuntsholing and its nearby locality only [11]. In 2016, a dengue outbreak was reported again in the endemic town of Phuntsholing, and in 2017 it was reported in the main towns of Samtse and Samdrup Jongkhar districts, which also share borders with India (Fig 1).

Until present, Bhutan has reported 3 serotypes of DENV circulating in the country; DENV-1, 2 and 3. DENV-2 and 3 were the most predominant serotypes during 2004 and 2005–2006 outbreaks, respectively [9]. However, a more recent report found DENV-1 as the most predominant serotype of DENV in Bhutan during 2013–2014 [11].

In this study, we performed molecular characterization of DENV from acute serum specimens collected from 2016 and 2017 outbreaks in order to elucidate the molecular epidemiology of the DENV in Bhutan and ultimately help in the development of dengue control strategies and appropriate interventions suitable to the affected districts in the country.

## Materials and methods

### Ethics statement

Specimens were collected at district hospitals during outbreaks and sent to RCDC, Bhutan for confirmation and further analysis, as mandated in the protocol for National Early Warning Alert and Response Surveillance (NEWARS; www.rcdc.gov.bt/WEB). Hence, no written consent was obtained from patients. A written permission was given to Armed Force Research Institute for Medical Sciences (AFRIMS, Bangkok, Thailand) by RCDC, Ministry of Health, Bhutan, for use of de-identified specimens for virus isolation and sequencing. Material was reviewed by the Walter Reed Army Institute of Research (WRAIR) and clearance was obtained for its presentation and/or publication.

### Specimen collection from outbreak sites

**Fig 2:**
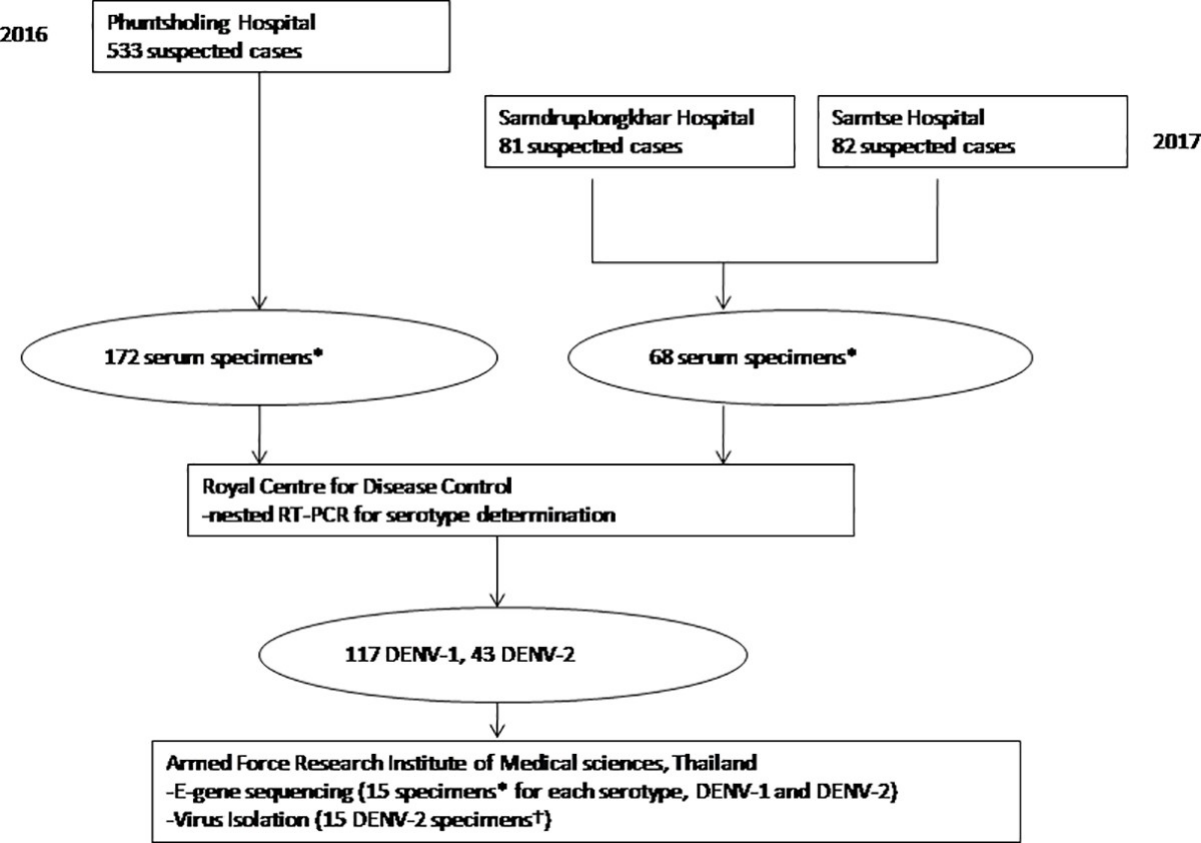
Flow chart demonstrating the process of specimen collection and analysis. *Randomly selected among samples having adequate volume. †Specimens from which E gene sequences could not be obtained directly.

Outbreaks of DF were reported from Phuntsholing in 2016, and Samtse and Samdrup Jongkhar in 2017 (Fig 1). As per the NEWARS protocol, DF is a reportable syndrome. Clinically suspected dengue was defined as fever (oral, rectal or axillary temperature >38°C), or history of fever lasting 2 to 7 days of unknown origin with two or more of the following: headache, retro-orbital pain, myalgia, arthralgia, rash, and hemorrhagic manifestation [6,12]. Acute blood specimens were collected from clinically suspected cases of DF in district hospitals of Phuntsholing, Samtse and Samdrup Jongkhar. A commercial rapid immunochromatographic test kit, SD BIOLINE Dengue IgG/IgM (Catalogue no. 11FK10) was used for initial confirmation of DENV infection. Samples that tested positive by rapid method, along with samples from clinically suspected cases were sent to RCDC to determine the infecting serotype by nested RT-PCR. Acute serum samples positive by nested RT-PCR were further sent to AFRIMS for investigation (Fig 2).

### Nested RT-PCR

Viral RNA was extracted from 140 μl of acute serum samples using QIAamp viral RNA mini kit (QIAGEN, Germany) following the manufacturer’s instructions. Nested RT-PCR was performed using a method modified from Lanciotti et al., 1992 as previously described [13,14]. One step RT-PCR was carried out using AMV reverse transcriptase (Promega, Madison, WI, USA) and AmpliTaq DNA polymerse (Life Technologies, USA) in the first round PCR. Nested PCR was performed using AmpliTaq DNA polymerase in the second round PCR.

### Virus isolation

Isolation of DENV was carried out with only the DENV nested RT-PCR positive acute serum samples from which the envelope gene sequences were not obtained directly. Virus isolation was performed to amplify viruses in samples with low viral load and facilitate sequencing. Specimens were inoculated into freshly prepared monolayers of C6/36 cells grown in Minimum Essential Medium (MEM, GIBCO) containing 10% heat inactivated fetal bovine serum (HIFBS), 1% Glutamine and 1% Penicillin and streptomycin. These cultures were maintained in maintenance medium (MM) containing RPMI with 5% HIFBS. A mock-infected C6/36 cell flask was included as a negative control. Cells underwent 3 passages and were observed for cytopathic effect (CPE). Identification of DENV serotypes was carried out by antigen capture ELISA as previously described [15,16]. Molecular confirmation of the isolates was performed by extracting DENV RNA from the cell culture supernatant followed by nested RT-PCR.

### Envelope (E) gene sequencing and phylogenetic analysis

Based on their volume adequacy, the DENV nested RT-PCR positive samples were selected for E gene sequencing. The first attempt was to obtain the full length of DENV E gene sequences directly from the DENV nested RT-PCR positive serum samples. The positive serum samples from which E gene sequences could not obtained directly were subjected for virus isolation. The obtained DENV isolates were used for E gene sequencing. E gene sequencing with acute serum samples and virus isolates was performed by either of the two methods- the capillary-based Sanger sequencing at AITbiotech Pvt Ltd. using DENV specific primers following the method previously described [11,17,18] or the Next Generation Sequencing (NGS) (Illumina MiSeq) using random primers and TruSeq RNA sample preparation kit (Illumina, USA) [19]. For Sanger sequencing, E gene fragment was amplified under the following thermocycler conditions; 48°C for 45 min, followed by 40 cycles of 94°C for 20 sec, 50°C for 30 sec, 72°C for 1 min and 1 cycle of 72°C for 7 min. For NGS, the DNA fragments were amplified during DNA library preparation under the following thermocycler conditions; 98°C for 10 sec, followed by 15 cycles of 98°C for 10 sec, 60°C for 30 sec, 72^o^c for 30 sec, and 1 cycle of 72°C for 5 min. Consensus sequences were generated by using Sequencher software (Gene Code Corp., USA) and ngs/mapper v1.2.4. [a Walter Reed Army Institute of Research Viral Disease Branch (WRAIR VBD) bioinformatics in-house developed software]. The GenBank accession numbers of E gene sequences obtained from this study are MK651217-MK651236.

Maximum likelihood trees were constructed from new DENV E gene sequences from Bhutan, along with some previous sequences obtained from GenBank, some global sequences and some vaccine candidate sequences using RaxML [21] with the GTR+G model as the best-fit model of nucleotide substitution determined by jModelTestv2 [20].

### Statistical analysis

Epi Info version 7 was used for age group analysis of suspected dengue cases.

## Results

### Descriptive analysis of outbreaks

**Fig 3:**
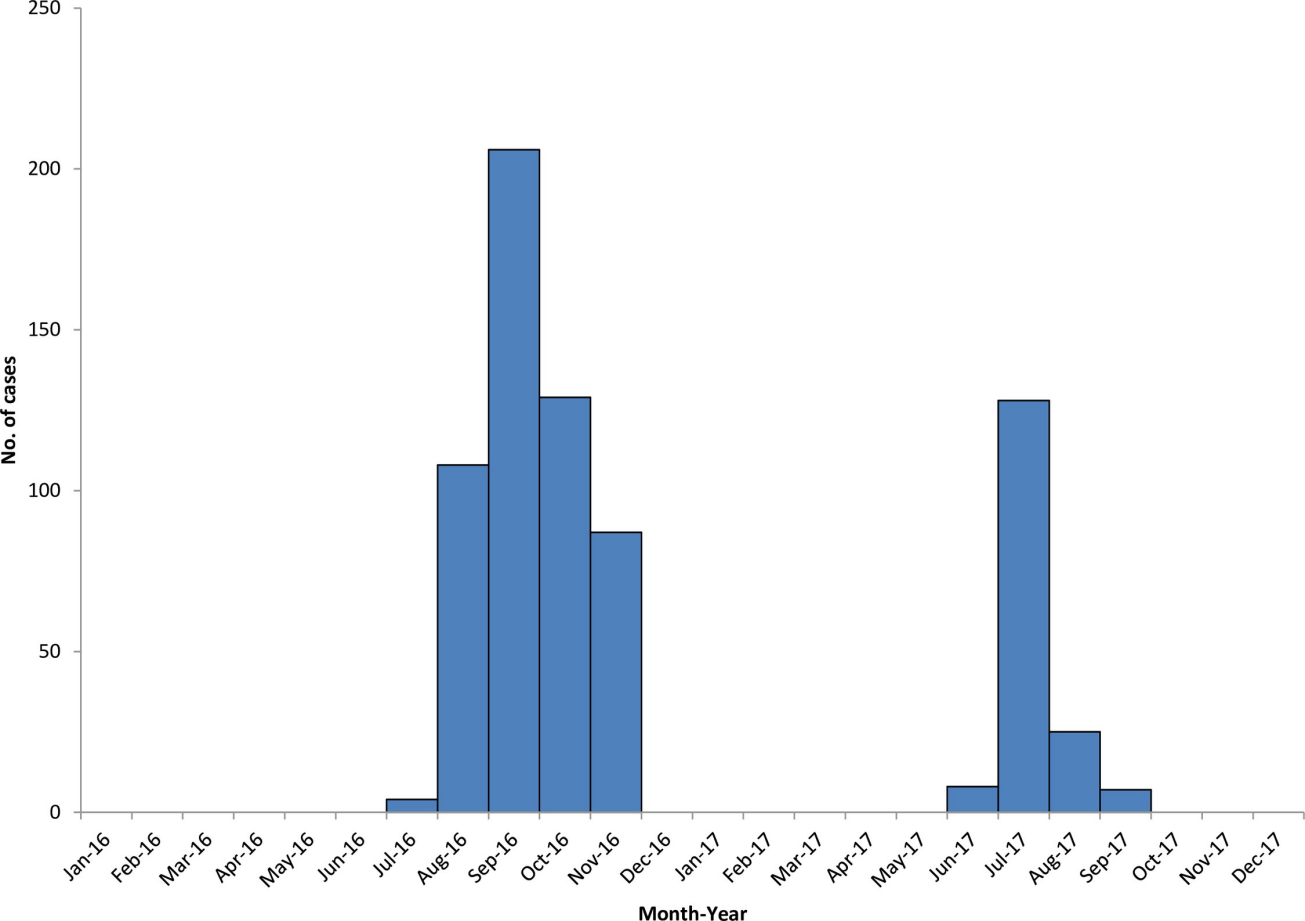
Epicurve of DF outbreaks that occurred in Phuntsholing (2016); Samtse and Samdrup Jongkhar (2017).

In Phuntsholing, the outbreak of DF occurred from July until November of 2016 while in 2017, it occurred during June until September in Samtse and Samdrup Jongkhar towns (Fig 3).

A total of 533 clinically suspected cases were recorded in Phuntsholing during the outbreak in 2016, and in 2017, 82 and 81 clinically suspected cases were recorded in Samtse and Samdrup Jongkhar respectively (S1 Table). Age of these cases ranged from <1 to 88 years. In all three outbreaks, young adults of 25–34 years were mostly affected (Table 1). The median age of the cases was 26 and 29 years for 2016 and 2017 respectively. The cases were higher among males compared to females (ratio of 1.2:1).

**Table 1:** Distribution of clinically suspected cases by age and gender in each site.

Age category (years)	Cases by site			Gender	
	Phuntsholing (n = 533)	Samtse (n = 82)	Samdrup Jongkhar (n = 81)	Male (n = 377)	Female (n = 319)
0–5	37 (6.9%)	2 (2.4)	4 (4.9)	23 (6.1)	20 (6.3)
6–14	77 (14.4%)	17 (20.7)	6 (7.4)	54 (14.3)	46 (14.4)
15–24	121(22.7%)	18 (22.0)	12 (14.8)	80 (21.2)	71 (22.3)
25–34	162 (30.4%)	15 (18.3)	28 (34.6)	108 (28.6)	97 (30.4)
35–44	84 (15.8%)	14 (17.1)	19 (23.5)	66 (17.5)	51 (16.0)
45–64	41 (7.7%)	15 (18.3)	12 (14.8)	39 (10.3)	29 (9.1)
65+	11 (2.0%)	1 (1.2)	0 (0.0)	7 (1.9)	5 (1.6)

### Nested RT-PCR

A total of 240 randomly selected acute serum specimens (172 from 2016, 68 from 2017) sent to RCDC were tested for serotype identification by DENV nested RT-PCR and 160 (67%) were positive (S2 Table). Serotype identification for 160 PCR positive samples revealed 117 DENV-1 (93 in 2016 and 24 in 2017) and 43 DENV-2 (37 in 2016 and 6 in 2017).

### E gene sequencing and virus Isolation

Among DENV nested RT-PCR positive acute serum samples with adequate volume available, 15 DENV-1 and 15 DENV-2 were selected for sequencing. We were able to obtain E gene sequences directly from all 15 DENV-1 serum samples but not from DENV-2 serum samples. To obtain E gene sequences from DENV-2 serum samples, the 15 DENV-2 samples were subjected for virus isolation. Only 5 DENV-2 were successfully isolated and used for E gene sequencing. We were able to obtain E gene sequences from all 5 DENV-2 isolates.

### Phylogenetic analysis

**Fig 4:**
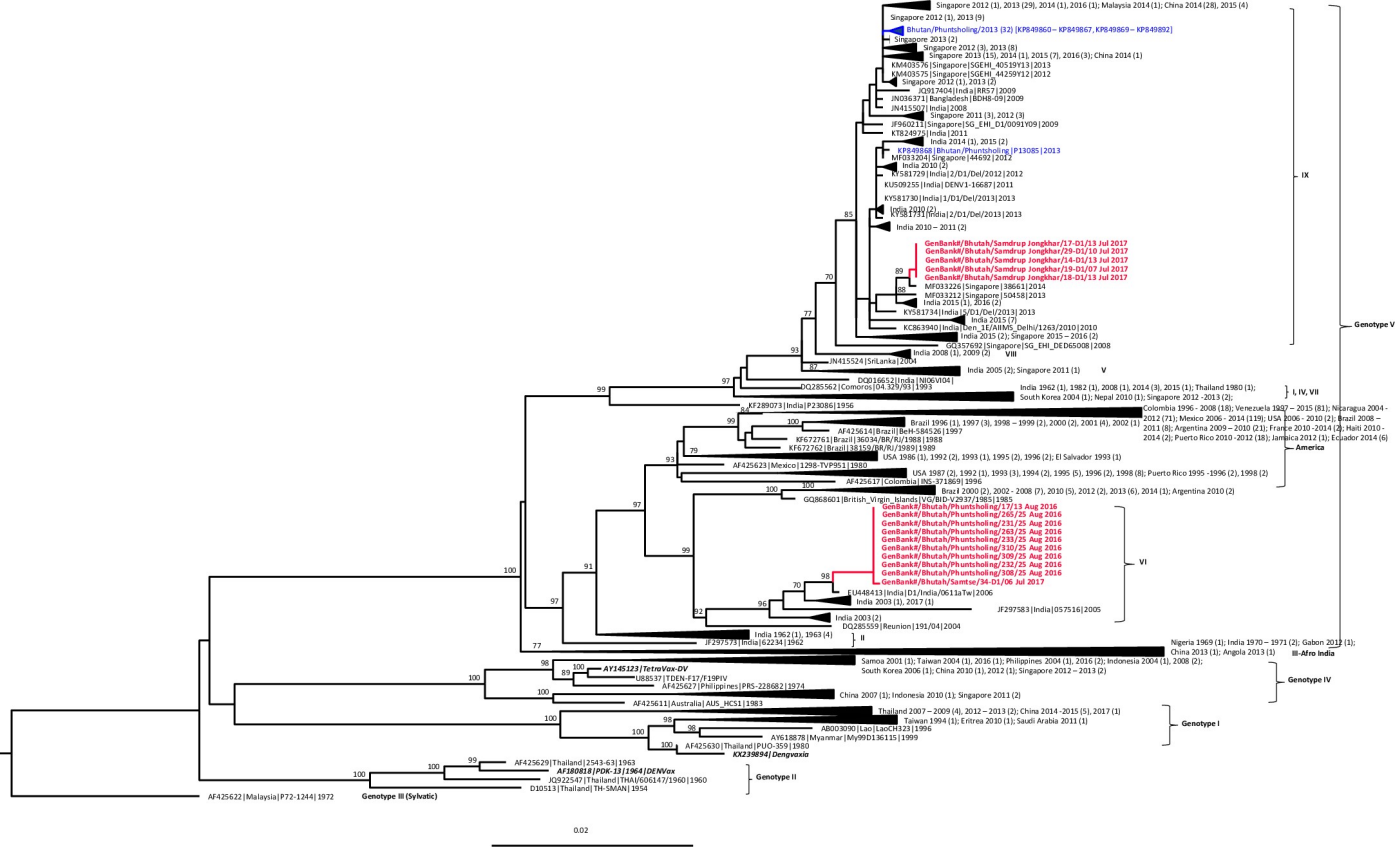
Phylogenetic tree. Maximum likelihood tree of 731 DENV-1 E gene sequences (1,485 bp) including 15 new sequences from this study (red bold), 33 sequences from previous study in Bhutan (blue), 3 vaccine strains sequences (black bold and italics), and 680 other sequences from GenBank (black). Bootstrap values are indicated at the major nodes.

The genotypes and clades of DENV circulating in Bhutan since 2004 to 2017 are summarized in in Table 2. GenBank accession numbers and other details of DENV sequences obtained from Bhutan during this study are given in S3 Table. ML tree for DENV-1 E gene was generated using 731 E gene sequences (1,485 bp), which included 15 sequences from Bhutan obtained during this study (red bold), 33 sequences obtained from previous studies in Bhutan [8] (blue), 680 global sequences (black), and 3 vaccine strain sequences including Dengvaxia, TetraVax-DV, and DENVax (black bold italics) (Fig 4). 680 global sequences used in this analysis included sequences from India previously analyzed by Dash et al., 2015 [23] and sequences with known DENV-1 genotypes from other countries randomly selected from the data available in GenBank on 8^th^ November 2018. All 15 new DENV-1 and 33 previous sequences from Bhutan belong to genotype V, as per the classification by Weaver et al, 2009 [22]. Within genotype V, the recent 2016/2017 Bhutan DENV sequences grouped separately into 2 clades. All of the sequences (9/9) from Phuntsholing, obtained in August 2016 and one sequence (1/1) from Samtse, obtained in July 2017 fell in Indian clade VI while five sequences (5/5), obtained in the month of July, 2017 fell in clade IX as per classification by Dash et. al. 2015 [23]. None of the vaccine strain sequences included in our phylogenetic tree fell within the same group as the Bhutan DENV-1.

**Table 2:** Genotypes and clades of Bhutan DENV 2004–2017 based on E gene sequences.

Year	DENV serotype	Genotype	Clade	Number of sequences	District	Reference
2004	DENV-2	Cosmopolitan	Not determined but closely related to Indian viruses from Indian subcontinent	2	Phuntsholing	Dorji et.al., 2009
2005	DENV-3	III	Not determined but closely related to Indian viruses from Indian subcontinent	19	Phuntsholing	
2006	DENV-3	III	Not determined but closely related to Indian viruses from Indian subcontinent		Phuntsholing	
2013	DENV-1	V	Indian IX	33	Phuntsholing	Zangmo et.al., 2015
2016	DENV-1	V	Indian VI	9	Phuntsholing	This study
2016	DENV-2	Cosmopolitan	Not determined but closely related to Indian virus from 2015	5	Phuntsholing	
2017	DENV-1	V	Indian VI	1	Samtse	
2017	DENV-1	V	Indian IX	5	Samdrup Jongkhar	

**Fig 5:**
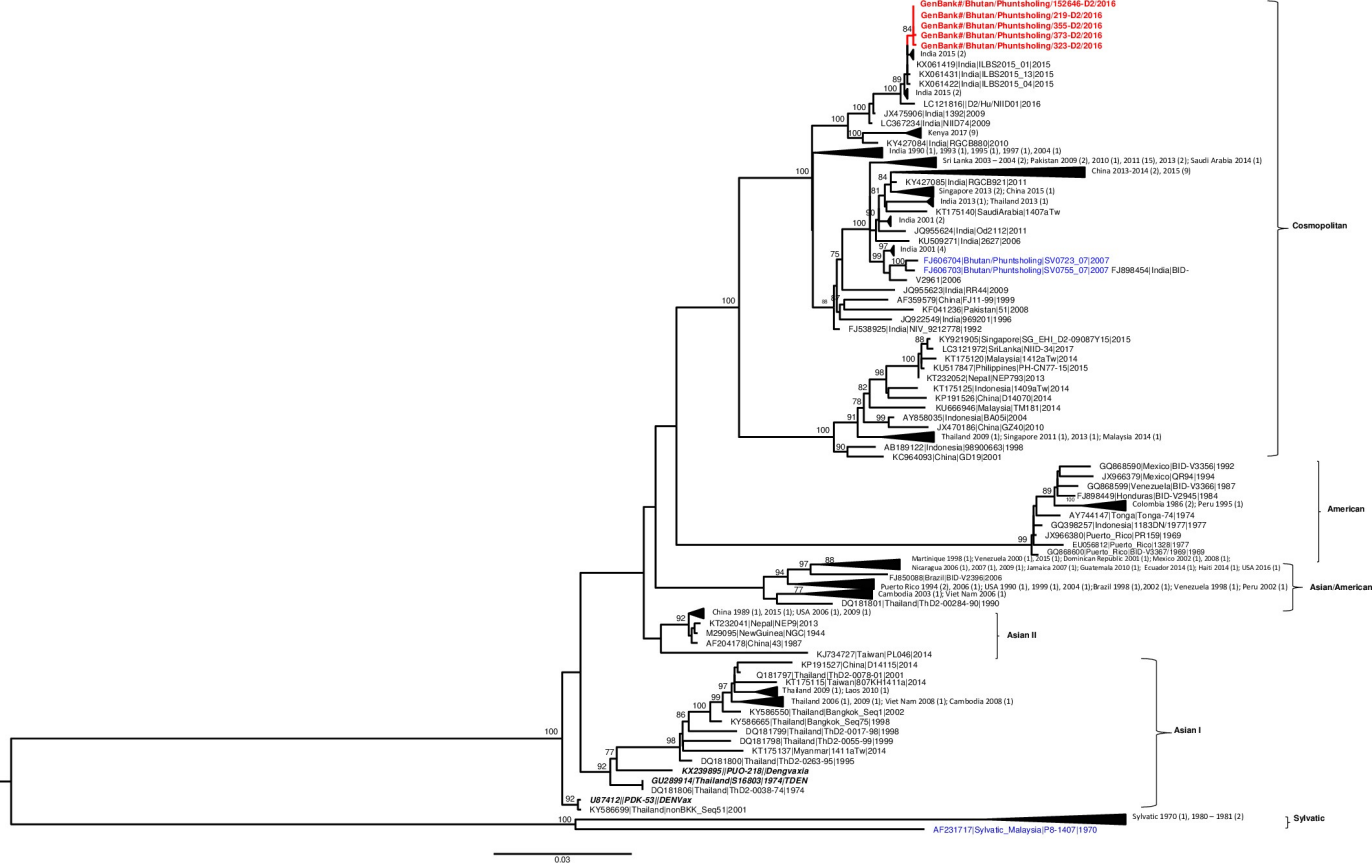
Phylogenetic tree. Maximum likelihood tree of 175 DENV-2 E gene sequences (1,485 bp) including 5 new sequences from this study (red bold), 2 sequences from previous study in Bhutan (blue), 3 vaccine strains sequences (black bold and italics), and 165 other sequences from GenBank (black). Bootstrap values are indicated at the major nodes.

Similarly, ML tree generated for DENV-2 E gene (Fig 5) using 5 new sequences obtained from this study (red bold), 2 sequences obtained from a previous study in Bhutan [8] (blue), 165 global sequences (black), and 3 vaccine strain sequences that include Dengvaxia, TDEN, and DENVax (black bold italics) obtained from GenBank. 165 global sequences used in this analysis include sequences from India and sequences with known DENV-2 genotypes from other countries randomly selected from data available in GenBank as on 8^th^ November 2018. All new sequences from Bhutan belong to the *Cosmopolitan* genotype as was the case in Bhutan in 2007 [8] and is closely related to sequences from India, 2015. Sequences obtained from the recent and past outbreaks were found to group in a separate clade.

## Discussion

Since the first outbreak of DF in 2004, Bhutan has reports of some sporadic cases and very few outbreaks [11]. Geographically, Bhutan is a mountainous country with most part of the country falling at an average height of 8000 ft above sea level. Nevertheless, incursion of tropical diseases into the low-lying areas in south have been noted in recent years [9,11,24]. As pointed out by studies, urbanization, globalization and lack of effective vector control, have probably contributed to the increasing epidemics of dengue and other tropical diseases in otherwise a low-risk country [10].

Here we are reporting outbreaks of DF from 3 places in southern Bhutan- Phuntsholing town under Chukha district (2016), and the main towns of Samtse and Samdrup Jongkhar districts (2017). This is also the first DF outbreak reported from Samdrup Jongkhar district. These 3 outbreaks occurred during the summer months of June-September of 2016 and 2017, as has been the case in past years [9,11]. Hot and humid climatic conditions during these months in the southern regions of Bhutan could also have contributed to dengue epidemics. Age groups affected during the 2016 and 2017 outbreaks were similar to those of previous outbreaks. High number of adults being affected is consistent with findings from studies in other countries where adults were found more likely to have clinical dengue than young children [25,26]. In Bhutan, this observation may also suggest that presence of dengue naïve individuals in the adult Bhutanese population, since these recent outbreaks were reported in places were dengue was either not previously or recently reported.

During the outbreaks of 2016 and 2017, we found DENV-1 as the predominating serotype circulating in the country followed by DENV-2. DENV-1 was first detected in Bhutan in 2006 and DENV-2 in 2004 [9]. However, DENV-3 was the predominating serotype during 2004 outbreak and DENV-2 in subsequent years (2005–06). There was no surveillance conducted during the years 2007–2012, and hence we have no information on dengue virology for these years. In 2013 and 2014, DENV-1 was the predominating serotype [11]. DENV-1 E gene sequences of 2016 and 2017 fell under genotype V, also known as the cosmopolitan genotype, the same genotype detected in 2013 and 2014. However, in this study Bhutanese DENV-1 isolates from Phuntsholing in 2016 and one isolate from Samtse in 2017 were located in the Indian clade VI which was closely related with the viruses that circulated in India in 2003 and 2006, but different from the Indian clade IX found in Bhutan in 2013. Meanwhile, most of the Bhutanese DENV-1 isolates from Samdrup Jongkhar in 2017 were grouped with the Indian clade IX that was the same clade previously found in Bhutan in 2013, and closely related to the viruses that circulated in India during 2015–2016. Without proper dengue surveillance, we can only speculate that the two distinct clades of DENV-1 genotype V found in 2016 and 2017 were rather imported from India, where they persist. The Indian clade IX could have been imported to Phuntsholing in 2013 and later to Samdrup Jongkhar in 2017, while the Indian clade VI was first imported to Phuntsholing in 2016 and later found in Samtse in 2017.

DENV-2 E gene sequences from Bhutan grouped with the cosmopolitan genotype, the same genotype as previous sequences found in 2007 in Bhutan. Within this genotype, sequences from the recent and past outbreaks grouped separately in clades. There is no information on sequences of DENV-2 obtained for the years between 2007 and 2016. Nevertheless, based on epidemiology and phylogenetic tree, it is likely that the strains for both the outbreaks were imported across the border.

Genotypes of DENV-1 (genotype V) and DENV-2 (cosmopolitan genotype) circulating in Bhutan were found different from the DENV-1 (genotype I) and DENV-2 (Asian I genotype) strains in the components of Dengvaxia vaccine, the first licensed tetravalent dengue vaccine, respectively. Previous study investigating the association between DENV genotypes and level of vaccine efficacy from the two phase III efficacy trials of the Dengvaxia vaccine including CYD14 trail in the Asia-Pacific region and CYD15 trial in Latin America, revealed the various association in each serotype [25]. Different level of vaccine efficacy against the three DENV-1 genotypes was not found. Among DENV-2 genotypes, the vaccine efficacy against Asian I genotype was significantly lower than that against the American-Asian and Cosmopolitan genotypes. Vaccine efficacy against DENV-4 genotype I was significantly lower than that against genotype II in participants of all ages, but among participants 9–16 years of age, the vaccine efficacy were similarly high between the two DENV-4 genotypes. For DENV-3, the association between vaccine efficacy and genotype was still uncertain [27]. Post-licensure research of this vaccine and other vaccines in the future is necessary to enhance understanding of this association. Continued investigation of DENV genetic epidemiology is critical to provide genetic background information of viral populations that would imply the vaccine efficacy, and benefit in providing and/or managing vaccine program in the country. We have several limitations in this study. The surveillance being a passive one, many pertinent demographic and epidemiological data could not be captured. We lack information on the days of infection (DOI) and details of symptoms presented. We were unable to establish the actual laboratory cases of the total suspected cases since due to lack of standard protocol and shortage of test kits for performing either IgM/gG or NS1 tests to screen all the cases. Thus we have no information on seroprevalence of DENV.

### Conclusion

The serotype and genotype of DENV isolated during the outbreaks of 2016 and 2017 in Bhutan closely resemble the Indian strains, suggesting that dengue outbreak in 2016 and 2017 was due to DENV imported from neighboring India. Such outbreaks of DF in newer places in Bhutan indicate a need for stronger dengue surveillance and vector control in the country.

Through this study we further identified certain gaps in the surveillance protocol for dengue in the country. The surveillance may need to be strengthened and stream-lined to have a well-planned study whereby epidemiological and virological data representing different geographic region, age group and other factors are well captured.

## Supporting information

S1 Table Dataset bearing age, sex, place and year of detection for suspected dengue cases. (XLSX)

S2 Table Laboratory results of samples subjected to PCR. (XLSX)

S3 Table Source modifiers table bearing gene accession numbers of samples subjected to sequencing. (XLSX)
